# Dichloridotetra­kis­(diniconazole)nickel(II)

**DOI:** 10.1107/S160053681103128X

**Published:** 2011-08-06

**Authors:** Zhi-Qiang Xiong, Xiu-Ying Song, Xu-Liang Nie

**Affiliations:** a Instrumental Analysis Center, Nanchang Hangkong University, Nanchang 330063, People’s Republic of China; b College of Sciences, Jiangxi Agricultural University, Nanchang 330045, People’s Republic of China

## Abstract

In the title compound, [NiCl_2_(C_15_H_17_Cl_2_N_3_O)_4_], the Ni atom lies on an inversion center and has an axially extended *trans*-NiCl_2_N_4_ octa­hedral geometry arising from its coordination by four diniconazole [systematic name: (*E*)-(*RS*)-1-(2,4-dichloro­phen­yl)-4,4-dimethyl-2-(1*H*-1,2,4-triazol-1-yl)pent-1-en-3-ol] ligands and two chloride ions. In the crystal, O—H⋯Cl hydrogen bonds link the mol­ecules into [100] chains.

## Related literature

For background to the use of diniconazole as a fungicide, see: Sumitomo Chemical (1984 ▶); Huang *et al.* (2003 ▶); Zhou *et al.* (2008 ▶). For further synthetic details, see: Fu (2002 ▶); Xia *et al.* (2001 ▶). For the isotypic zinc complex, see: Gao *et al.* (2001 ▶). For our previous work based on diniconazole, see: Xiong *et al.* (2010 ▶).
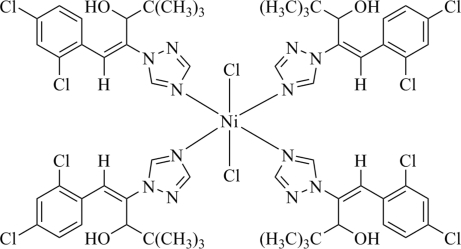


## Experimental

### 

#### Crystal data

[NiCl_2_(C_15_H_17_Cl_2_N_3_O)_4_]
*M* *_r_* = 1434.47
Triclinic, 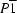

*a* = 8.7598 (6) Å
*b* = 13.7800 (9) Å
*c* = 15.1344 (10) Å
α = 90.672 (1)°
β = 98.521 (1)°
γ = 106.743 (1)°
*V* = 1727.3 (2) Å^3^
*Z* = 1
Mo *K*α radiation
μ = 0.72 mm^−1^
*T* = 296 K
0.25 × 0.22 × 0.21 mm

#### Data collection

Bruker APEXII CCD diffractometer
Absorption correction: multi-scan (*SADABS*; Sheldrick, 2007 ▶) *T*_min_ = 0.840, *T*_max_ = 0.863
13440 measured reflections
6383 independent reflections
4766 reflections with *I* > 2σ(*I*)
*R*_int_ = 0.025

#### Refinement

*R*[*F* ^2^ > 2σ(*F* ^2^)] = 0.041
*wR*(*F* ^2^) = 0.104
*S* = 1.02
6383 reflections
402 parameters
H-atom parameters constrained
Δρ_max_ = 0.35 e Å^−3^
Δρ_min_ = −0.42 e Å^−3^

### 

Data collection: *APEX2* (Bruker, 2007 ▶); cell refinement: *SAINT* (Bruker, 2007 ▶); data reduction: *SAINT*; program(s) used to solve structure: *SHELXS97* (Sheldrick, 2008 ▶); program(s) used to refine structure: *SHELXL97* (Sheldrick, 2008 ▶); molecular graphics: *SHELXTL* (Sheldrick, 2008 ▶); software used to prepare material for publication: *SHELXTL*.

## Supplementary Material

Crystal structure: contains datablock(s) I, global. DOI: 10.1107/S160053681103128X/hb6336sup1.cif

Structure factors: contains datablock(s) I. DOI: 10.1107/S160053681103128X/hb6336Isup2.hkl

Additional supplementary materials:  crystallographic information; 3D view; checkCIF report

## Figures and Tables

**Table 1:** Selected bond lengths (Å)

Ni1—N1	2.091 (2)
Ni1—N4	2.106 (2)
Ni1—Cl1	2.4860 (6)

**Table 2:** Hydrogen-bond geometry (Å, °)

*D*—H⋯*A*	*D*—H	H⋯*A*	*D*⋯*A*	*D*—H⋯*A*
O1—H1*A*⋯Cl1^i^	0.82	2.36	3.1460 (19)	160
O2—H2*A*⋯Cl1^i^	0.82	2.32	3.123 (2)	169

Symmetry code: (i) 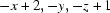.

**Fig. 1.**
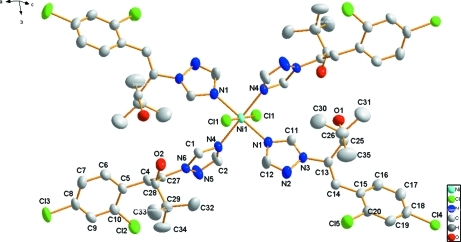
The molecular structure of (I), showing displacement ellipsoids at the 40% probability level.

**Fig. 2.**
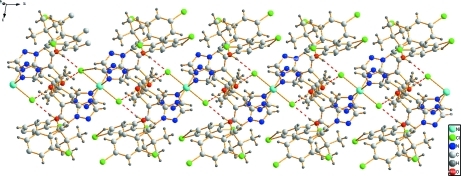
The crystal packing of (I), showing a hydrogen-bonded chain; H-bonds are shown as dashed lines.

## supplementary crystallographic information

### Comment

Diniconazole [(*E*)-(*RS*)-1-(2,4-dichlorophenyl)-4,4-dimethyl-2- (1*H*-1,2,4-triazol-1-yl)-pent-1-en-3-ol] is a highly active triazole fungicide (Sumitomo Chemical, 1984). It is widely used for control of a broad range of fungal diseases in many crops, such as corn, wheat, peanut, grape and apple (Huang *et al.*, 2003; Zhou *et al.*, 2008). Because of its strong antimicrobial activities and its wide applications, the synthesis of diniconazole (Fu *et al.*, 2002; Xia *et al.*, 2001) and its salts (Gao *et al.*, 2001) have attracted much attention. Recently, our group have reported the crystal structure of diniconazole (Xiong *et al.*, 2010). In this paper, we report the synthesis and crystal structure of a new nickel(II) complex, (I), incorporating diniconazole.

The asymmetric unit of the title compound, [Ni(C_15_H_17_Cl_2_N_3_O) _4_Cl_2_], consists of one nickel(II) ion, two diniconazole ligands and one coordinated chloride ion. The Ni atom lies on an inversion center and has a slightly distorted octahedral geometry. The equatorial positions are occupied by four N atoms from four (E)-(*RS*)-1-(2,4- dichlorophenyl)-4,4-dimethyl-2-(1*H*-1,2,4-triazol-1-yl)-pent-1-en-3-ol ligands. The axial sites are occupied by two Cl atoms(Fig. 1). The Ni—N distance are 2.123 (3) and 2.147 (3) Å and Ni—Cl is 2.5222 (9) Å. In the crystal packing, intermolecular O—H···Cl hydrogen bonds (Table 1) link the molecules into chains along the *a* axis(Fig. 2). The structure of the title compound is isostructural to previously reported zinc (II) complexe constructed by Zn^2+^ and diniconazole (Gao *et al.*, 2001).

### Experimental

NiCl_2_.6H_2_O (0.024 g, 0.1 mmol) was dissolved in ethanol (10 ml), and diniconazole(0.063 g, 0.2 mmol) was dissolved in ethanol (10 ml). The NiCl_2_ solution was added to the diniconazole solution slowly under stirring. The mixture were filtered after stirring for 1 h. Green blocks of (I) were obtained by slow concentration of an ethanol solution. Anal. Calcd. For C_60_H_68_Cl_10_NiN_12_O_4_ (%) (Mr = 1434.48): C, 50.24; H, 4.78; N, 11.72. Found (%): C, 55.21; H, 4.80; N, 11.70.

### Refinement

All H atoms were included in calculated positions and refined as riding atoms, with C—H = 0.93–0.96 Å, O—H = 0.82 Å, with *U*_iso_(H) = 1.5 *U*_eq_(C) for methyl H atoms and 1.2 *U*_eq_(C) for all other H atoms.

### Crystal data

**Table:**

[NiCl_2_(C_15_H_17_Cl_2_N_3_O)_4_]	*Z* = 1
*M**_r_* = 1434.47	*F*(000) = 742
Triclinic, *P*1	*D*_x_ = 1.379 Mg m^−^^3^
Hall symbol: -P 1	Mo *K*α radiation, λ = 0.71073 Å
*a* = 8.7598 (6) Å	Cell parameters from 3863 reflections
*b* = 13.7800 (9) Å	θ = 2.5–26.1°
*c* = 15.1344 (10) Å	µ = 0.72 mm^−^^1^
α = 90.672 (1)°	*T* = 296 K
β = 98.521 (1)°	Block, green
γ = 106.743 (1)°	0.25 × 0.22 × 0.21 mm
*V* = 1727.3 (2) Å^3^	

### Data collection

**Table:**

Bruker APEXII CCD diffractometer	6383 independent reflections
Radiation source: fine-focus sealed tube	4766 reflections with *I* > 2σ(*I*)
graphite	*R*_int_ = 0.025
φ and ω scans	θ_max_ = 25.5°, θ_min_ = 2.5°
Absorption correction: multi-scan (*SADABS*; Sheldrick, 2007)	*h* = −10→10
*T*_min_ = 0.840, *T*_max_ = 0.863	*k* = −16→16
13440 measured reflections	*l* = −18→18

### Refinement

**Table:**

Refinement on *F*^2^	Primary atom site location: structure-invariant direct methods
Least-squares matrix: full	Secondary atom site location: difference Fourier map
*R*[*F*^2^ > 2σ(*F*^2^)] = 0.041	Hydrogen site location: inferred from neighbouring sites
*wR*(*F*^2^) = 0.104	H-atom parameters constrained
*S* = 1.02	*w* = 1/[σ^2^(*F*_o_^2^) + (0.0497*P*)^2^ + 0.4285*P*] where *P* = (*F*_o_^2^ + 2*F*_c_^2^)/3
6383 reflections	(Δ/σ)_max_ = 0.001
402 parameters	Δρ_max_ = 0.35 e Å^−^^3^
0 restraints	Δρ_min_ = −0.42 e Å^−^^3^

### Special details

**Table:**

Geometry. All e.s.d.'s (except the e.s.d. in the dihedral angle between two l.s. planes) are estimated using the full covariance matrix. The cell e.s.d.'s are taken into account individually in the estimation of e.s.d.'s in distances, angles and torsion angles; correlations between e.s.d.'s in cell parameters are only used when they are defined by crystal symmetry. An approximate (isotropic) treatment of cell e.s.d.'s is used for estimating e.s.d.'s involving l.s. planes.
Refinement. Refinement of *F*^2^ against ALL reflections. The weighted *R*-factor *wR* and goodness of fit *S* are based on *F*^2^, conventional *R*-factors *R* are based on *F*, with *F* set to zero for negative *F*^2^. The threshold expression of *F*^2^ > σ(*F*^2^) is used only for calculating *R*-factors(gt) *etc*. and is not relevant to the choice of reflections for refinement. *R*-factors based on *F*^2^ are statistically about twice as large as those based on *F*, and *R*- factors based on ALL data will be even larger.

### Fractional atomic coordinates and isotropic or equivalent isotropic displacement parameters (Å^2^)

**Table:**

	*x*	*y*	*z*	*U*_iso_*/*U*_eq_	
Ni1	0.5000	0.0000	0.5000	0.02802 (13)	
Cl1	0.73567 (8)	−0.02454 (5)	0.60068 (4)	0.04154 (18)	
Cl5	0.58239 (9)	−0.34451 (7)	0.02533 (5)	0.0630 (2)	
Cl2	1.11706 (10)	0.52902 (6)	0.24982 (6)	0.0638 (2)	
Cl3	1.66829 (9)	0.47117 (7)	0.18141 (6)	0.0634 (2)	
Cl4	1.16064 (13)	−0.36651 (8)	−0.03016 (7)	0.0854 (3)	
N4	0.6518 (2)	0.13360 (16)	0.45770 (14)	0.0341 (5)	
N3	0.6487 (2)	−0.12645 (16)	0.28494 (13)	0.0332 (5)	
N1	0.5419 (2)	−0.08273 (16)	0.39414 (13)	0.0347 (5)	
N6	0.8613 (2)	0.23450 (15)	0.41014 (13)	0.0316 (5)	
C2	0.6135 (3)	0.2041 (2)	0.4058 (2)	0.0512 (8)	
H2	0.5084	0.2081	0.3926	0.061*	
C12	0.4329 (3)	−0.1519 (2)	0.33470 (19)	0.0498 (8)	
H12	0.3248	−0.1765	0.3412	0.060*	
C11	0.6754 (3)	−0.0703 (2)	0.36072 (16)	0.0351 (6)	
H11	0.7757	−0.0278	0.3866	0.042*	
C20	0.7892 (3)	−0.3141 (2)	0.06064 (17)	0.0404 (7)	
C16	1.0225 (3)	−0.2331 (2)	0.16512 (18)	0.0419 (7)	
H16	1.0712	−0.1945	0.2181	0.050*	
N2	0.4896 (3)	−0.1819 (2)	0.26754 (15)	0.0504 (6)	
C13	0.7614 (3)	−0.1355 (2)	0.22698 (15)	0.0317 (6)	
C14	0.7522 (3)	−0.2281 (2)	0.19829 (16)	0.0374 (6)	
H14	0.6722	−0.2810	0.2164	0.045*	
C15	0.8564 (3)	−0.25637 (19)	0.14017 (16)	0.0352 (6)	
C19	0.8817 (4)	−0.3476 (2)	0.00823 (19)	0.0485 (7)	
H19	0.8341	−0.3863	−0.0448	0.058*	
C17	1.1169 (4)	−0.2659 (2)	0.1134 (2)	0.0478 (7)	
H17	1.2281	−0.2494	0.1311	0.057*	
C18	1.0450 (4)	−0.3230 (2)	0.0357 (2)	0.0493 (8)	
C1	0.8086 (3)	0.15564 (19)	0.45978 (16)	0.0336 (6)	
H1	0.8741	0.1211	0.4914	0.040*	
C27	1.0189 (3)	0.27541 (18)	0.38578 (16)	0.0298 (5)	
C8	1.4775 (3)	0.4233 (2)	0.21011 (17)	0.0405 (7)	
C9	1.3818 (3)	0.4858 (2)	0.21392 (17)	0.0421 (7)	
H9	1.4153	0.5525	0.1973	0.051*	
C5	1.1815 (3)	0.3492 (2)	0.26917 (16)	0.0342 (6)	
C7	1.4256 (3)	0.3228 (2)	0.23023 (18)	0.0463 (7)	
H7	1.4885	0.2798	0.2243	0.056*	
C4	1.0282 (3)	0.31053 (19)	0.30519 (17)	0.0355 (6)	
H4	0.9329	0.3110	0.2690	0.043*	
C6	1.2785 (3)	0.2874 (2)	0.25922 (18)	0.0426 (7)	
H6	1.2432	0.2197	0.2726	0.051*	
N5	0.7347 (3)	0.26722 (19)	0.37477 (18)	0.0533 (7)	
C10	1.2342 (3)	0.4480 (2)	0.24291 (17)	0.0380 (6)	
C28	1.1612 (3)	0.2793 (2)	0.45744 (17)	0.0352 (6)	
H28	1.2570	0.2937	0.4280	0.042*	
C25	0.8684 (3)	−0.0378 (2)	0.19969 (16)	0.0358 (6)	
H25	0.9532	−0.0544	0.1725	0.043*	
C29	1.1960 (3)	0.3618 (2)	0.53325 (18)	0.0428 (7)	
C26	0.7828 (4)	0.0188 (2)	0.13069 (19)	0.0500 (8)	
O2	1.1449 (2)	0.18368 (14)	0.49624 (13)	0.0449 (5)	
H2A	1.1757	0.1469	0.4644	0.067*	
O1	0.9460 (2)	0.03079 (14)	0.27532 (12)	0.0452 (5)	
H1A	1.0243	0.0149	0.3002	0.068*	
C30	0.6677 (4)	0.0650 (3)	0.1709 (3)	0.0728 (11)	
H30A	0.6340	0.1104	0.1300	0.109*	
H30B	0.7220	0.1019	0.2264	0.109*	
H30C	0.5750	0.0119	0.1816	0.109*	
C31	0.9151 (5)	0.1044 (3)	0.0990 (3)	0.0811 (12)	
H31A	0.8668	0.1393	0.0535	0.122*	
H31B	0.9895	0.0762	0.0749	0.122*	
H31C	0.9716	0.1513	0.1486	0.122*	
C32	1.0688 (4)	0.3386 (3)	0.5958 (2)	0.0628 (9)	
H32A	1.0568	0.2719	0.6171	0.094*	
H32B	0.9674	0.3418	0.5637	0.094*	
H32C	1.1027	0.3877	0.6457	0.094*	
C33	1.3603 (4)	0.3659 (3)	0.5870 (2)	0.0716 (10)	
H33A	1.3912	0.4200	0.6325	0.107*	
H33B	1.4397	0.3775	0.5478	0.107*	
H33C	1.3532	0.3026	0.6145	0.107*	
C34	1.2064 (5)	0.4639 (2)	0.4925 (2)	0.0731 (11)	
H34A	1.2360	0.5165	0.5394	0.110*	
H34B	1.1034	0.4617	0.4589	0.110*	
H34C	1.2863	0.4777	0.4536	0.110*	
C35	0.6923 (5)	−0.0543 (3)	0.0510 (2)	0.0822 (12)	
H35A	0.6015	−0.1035	0.0687	0.123*	
H35B	0.7633	−0.0882	0.0307	0.123*	
H35C	0.6552	−0.0171	0.0035	0.123*	

### Atomic displacement parameters (Å^2^)

**Table:**

	*U*^11^	*U*^22^	*U*^33^	*U*^12^	*U*^13^	*U*^23^
Ni1	0.0224 (2)	0.0333 (3)	0.0295 (2)	0.00732 (19)	0.00932 (17)	0.00260 (19)
Cl1	0.0324 (3)	0.0500 (4)	0.0446 (4)	0.0167 (3)	0.0042 (3)	0.0057 (3)
Cl5	0.0451 (4)	0.0772 (6)	0.0583 (5)	0.0079 (4)	0.0038 (4)	−0.0208 (4)
Cl2	0.0602 (5)	0.0569 (5)	0.0886 (6)	0.0290 (4)	0.0312 (4)	0.0276 (4)
Cl3	0.0354 (4)	0.0843 (6)	0.0651 (5)	0.0013 (4)	0.0242 (4)	−0.0009 (4)
Cl4	0.0851 (7)	0.0949 (8)	0.0974 (7)	0.0448 (6)	0.0457 (6)	−0.0131 (6)
N4	0.0270 (11)	0.0379 (13)	0.0384 (12)	0.0075 (10)	0.0125 (9)	0.0064 (10)
N3	0.0280 (11)	0.0401 (13)	0.0317 (11)	0.0084 (10)	0.0082 (9)	−0.0035 (9)
N1	0.0302 (12)	0.0422 (14)	0.0333 (11)	0.0115 (10)	0.0088 (9)	0.0007 (10)
N6	0.0273 (11)	0.0302 (12)	0.0371 (11)	0.0058 (9)	0.0098 (9)	0.0066 (9)
C2	0.0283 (15)	0.055 (2)	0.075 (2)	0.0145 (14)	0.0176 (14)	0.0283 (16)
C12	0.0275 (15)	0.066 (2)	0.0526 (18)	0.0065 (14)	0.0116 (13)	−0.0127 (15)
C11	0.0291 (14)	0.0413 (16)	0.0339 (14)	0.0077 (12)	0.0069 (11)	−0.0045 (11)
C20	0.0421 (16)	0.0396 (17)	0.0404 (15)	0.0102 (13)	0.0129 (12)	−0.0014 (12)
C16	0.0440 (16)	0.0460 (17)	0.0378 (15)	0.0177 (14)	0.0044 (12)	−0.0013 (12)
N2	0.0315 (13)	0.0666 (18)	0.0470 (14)	0.0047 (12)	0.0079 (11)	−0.0173 (12)
C13	0.0296 (13)	0.0413 (16)	0.0248 (12)	0.0118 (12)	0.0038 (10)	−0.0022 (11)
C14	0.0404 (15)	0.0387 (16)	0.0324 (14)	0.0074 (12)	0.0123 (11)	−0.0006 (12)
C15	0.0451 (16)	0.0315 (15)	0.0326 (13)	0.0129 (12)	0.0142 (12)	0.0010 (11)
C19	0.061 (2)	0.0440 (18)	0.0421 (16)	0.0139 (15)	0.0161 (14)	−0.0082 (13)
C17	0.0461 (17)	0.0509 (19)	0.0548 (18)	0.0239 (15)	0.0148 (14)	0.0080 (15)
C18	0.059 (2)	0.0460 (19)	0.0553 (19)	0.0262 (16)	0.0273 (16)	0.0052 (15)
C1	0.0273 (13)	0.0353 (15)	0.0366 (14)	0.0048 (11)	0.0084 (11)	0.0070 (11)
C27	0.0269 (13)	0.0253 (14)	0.0364 (13)	0.0043 (10)	0.0099 (10)	0.0022 (10)
C8	0.0294 (14)	0.0551 (19)	0.0337 (14)	0.0043 (13)	0.0105 (11)	0.0009 (13)
C9	0.0400 (16)	0.0408 (17)	0.0393 (15)	−0.0006 (13)	0.0113 (12)	0.0054 (12)
C5	0.0304 (14)	0.0399 (16)	0.0308 (13)	0.0060 (12)	0.0089 (11)	0.0051 (11)
C7	0.0430 (17)	0.058 (2)	0.0441 (16)	0.0196 (15)	0.0153 (13)	0.0031 (14)
C4	0.0272 (13)	0.0376 (16)	0.0386 (14)	0.0034 (11)	0.0076 (11)	0.0067 (12)
C6	0.0459 (17)	0.0371 (16)	0.0450 (16)	0.0088 (13)	0.0146 (13)	0.0082 (12)
N5	0.0329 (13)	0.0535 (16)	0.0797 (18)	0.0164 (12)	0.0186 (12)	0.0329 (14)
C10	0.0364 (15)	0.0428 (17)	0.0353 (14)	0.0111 (12)	0.0085 (11)	0.0072 (12)
C28	0.0295 (13)	0.0389 (16)	0.0396 (14)	0.0098 (12)	0.0128 (11)	0.0078 (12)
C25	0.0342 (14)	0.0384 (16)	0.0368 (14)	0.0118 (12)	0.0097 (11)	−0.0013 (11)
C29	0.0407 (16)	0.0423 (17)	0.0405 (15)	0.0065 (13)	0.0030 (12)	−0.0013 (13)
C26	0.062 (2)	0.0432 (18)	0.0439 (16)	0.0164 (15)	0.0028 (14)	0.0049 (13)
O2	0.0480 (12)	0.0463 (12)	0.0510 (12)	0.0247 (10)	0.0186 (9)	0.0152 (9)
O1	0.0369 (11)	0.0462 (12)	0.0479 (11)	0.0091 (9)	−0.0006 (9)	−0.0080 (9)
C30	0.077 (3)	0.068 (3)	0.082 (3)	0.041 (2)	0.002 (2)	0.014 (2)
C31	0.102 (3)	0.064 (2)	0.078 (3)	0.017 (2)	0.026 (2)	0.028 (2)
C32	0.065 (2)	0.074 (2)	0.0489 (18)	0.0181 (18)	0.0136 (16)	−0.0163 (16)
C33	0.049 (2)	0.095 (3)	0.056 (2)	0.0064 (19)	−0.0071 (16)	−0.0044 (19)
C34	0.095 (3)	0.040 (2)	0.072 (2)	0.0069 (18)	0.002 (2)	−0.0093 (17)
C35	0.116 (3)	0.071 (3)	0.050 (2)	0.029 (2)	−0.020 (2)	0.0037 (18)

### Geometric parameters (Å, °)

**Table:**

Ni1—N1^i^	2.091 (2)	C8—C7	1.380 (4)
Ni1—N1	2.091 (2)	C9—C10	1.386 (4)
Ni1—N4	2.106 (2)	C9—H9	0.9300
Ni1—N4^i^	2.106 (2)	C5—C6	1.386 (4)
Ni1—Cl1^i^	2.4860 (6)	C5—C10	1.389 (4)
Ni1—Cl1	2.4860 (6)	C5—C4	1.481 (3)
Cl5—C20	1.736 (3)	C7—C6	1.378 (4)
Cl2—C10	1.732 (3)	C7—H7	0.9300
Cl3—C8	1.733 (3)	C4—H4	0.9300
Cl4—C18	1.737 (3)	C6—H6	0.9300
N4—C1	1.314 (3)	C28—O2	1.426 (3)
N4—C2	1.343 (3)	C28—C29	1.539 (4)
N3—C11	1.328 (3)	C28—H28	0.9800
N3—N2	1.367 (3)	C25—O1	1.428 (3)
N3—C13	1.444 (3)	C25—C26	1.545 (4)
N1—C11	1.310 (3)	C25—H25	0.9800
N1—C12	1.353 (3)	C29—C34	1.527 (4)
N6—C1	1.338 (3)	C29—C33	1.531 (4)
N6—N5	1.355 (3)	C29—C32	1.534 (4)
N6—C27	1.438 (3)	C26—C35	1.525 (4)
C2—N5	1.315 (3)	C26—C30	1.528 (5)
C2—H2	0.9300	C26—C31	1.535 (4)
C12—N2	1.307 (3)	O2—H2A	0.8200
C12—H12	0.9300	O1—H1A	0.8200
C11—H11	0.9300	C30—H30A	0.9600
C20—C19	1.377 (4)	C30—H30B	0.9600
C20—C15	1.387 (4)	C30—H30C	0.9600
C16—C17	1.378 (4)	C31—H31A	0.9600
C16—C15	1.389 (4)	C31—H31B	0.9600
C16—H16	0.9300	C31—H31C	0.9600
C13—C14	1.319 (4)	C32—H32A	0.9600
C13—C25	1.504 (4)	C32—H32B	0.9600
C14—C15	1.481 (3)	C32—H32C	0.9600
C14—H14	0.9300	C33—H33A	0.9600
C19—C18	1.370 (4)	C33—H33B	0.9600
C19—H19	0.9300	C33—H33C	0.9600
C17—C18	1.368 (4)	C34—H34A	0.9600
C17—H17	0.9300	C34—H34B	0.9600
C1—H1	0.9300	C34—H34C	0.9600
C27—C4	1.323 (3)	C35—H35A	0.9600
C27—C28	1.514 (3)	C35—H35B	0.9600
C8—C9	1.371 (4)	C35—H35C	0.9600
			
N1^i^—Ni1—N1	180.0	C6—C7—C8	118.7 (3)
N1^i^—Ni1—N4	90.43 (8)	C6—C7—H7	120.7
N1—Ni1—N4	89.57 (8)	C8—C7—H7	120.7
N1^i^—Ni1—N4^i^	89.57 (8)	C27—C4—C5	123.9 (2)
N1—Ni1—N4^i^	90.43 (8)	C27—C4—H4	118.1
N4—Ni1—N4^i^	180.00 (9)	C5—C4—H4	118.1
N1^i^—Ni1—Cl1^i^	91.80 (6)	C7—C6—C5	122.4 (3)
N1—Ni1—Cl1^i^	88.20 (6)	C7—C6—H6	118.8
N4—Ni1—Cl1^i^	90.45 (6)	C5—C6—H6	118.8
N4^i^—Ni1—Cl1^i^	89.55 (6)	C2—N5—N6	102.3 (2)
N1^i^—Ni1—Cl1	88.20 (6)	C9—C10—C5	122.0 (3)
N1—Ni1—Cl1	91.80 (6)	C9—C10—Cl2	118.2 (2)
N4—Ni1—Cl1	89.55 (6)	C5—C10—Cl2	119.8 (2)
N4^i^—Ni1—Cl1	90.45 (6)	O2—C28—C27	111.6 (2)
Cl1^i^—Ni1—Cl1	180.000 (1)	O2—C28—C29	108.5 (2)
C1—N4—C2	102.5 (2)	C27—C28—C29	115.3 (2)
C1—N4—Ni1	126.49 (17)	O2—C28—H28	107.0
C2—N4—Ni1	129.53 (17)	C27—C28—H28	107.0
C11—N3—N2	109.2 (2)	C29—C28—H28	107.0
C11—N3—C13	129.2 (2)	O1—C25—C13	111.5 (2)
N2—N3—C13	121.6 (2)	O1—C25—C26	108.0 (2)
C11—N1—C12	102.3 (2)	C13—C25—C26	114.9 (2)
C11—N1—Ni1	128.67 (17)	O1—C25—H25	107.4
C12—N1—Ni1	128.40 (17)	C13—C25—H25	107.4
C1—N6—N5	109.0 (2)	C26—C25—H25	107.4
C1—N6—C27	128.9 (2)	C34—C29—C33	109.3 (3)
N5—N6—C27	121.71 (19)	C34—C29—C32	110.2 (3)
N5—C2—N4	115.4 (2)	C33—C29—C32	109.0 (2)
N5—C2—H2	122.3	C34—C29—C28	109.1 (2)
N4—C2—H2	122.3	C33—C29—C28	106.4 (2)
N2—C12—N1	115.6 (2)	C32—C29—C28	112.7 (2)
N2—C12—H12	122.2	C35—C26—C30	110.5 (3)
N1—C12—H12	122.2	C35—C26—C31	109.0 (3)
N1—C11—N3	111.1 (2)	C30—C26—C31	108.9 (3)
N1—C11—H11	124.5	C35—C26—C25	109.1 (2)
N3—C11—H11	124.5	C30—C26—C25	112.3 (2)
C19—C20—C15	122.1 (3)	C31—C26—C25	107.0 (3)
C19—C20—Cl5	118.7 (2)	C28—O2—H2A	109.5
C15—C20—Cl5	119.2 (2)	C25—O1—H1A	109.5
C17—C16—C15	121.6 (3)	C26—C30—H30A	109.5
C17—C16—H16	119.2	C26—C30—H30B	109.5
C15—C16—H16	119.2	H30A—C30—H30B	109.5
C12—N2—N3	101.9 (2)	C26—C30—H30C	109.5
C14—C13—N3	116.9 (2)	H30A—C30—H30C	109.5
C14—C13—C25	126.6 (2)	H30B—C30—H30C	109.5
N3—C13—C25	116.4 (2)	C26—C31—H31A	109.5
C13—C14—C15	126.6 (2)	C26—C31—H31B	109.5
C13—C14—H14	116.7	H31A—C31—H31B	109.5
C15—C14—H14	116.7	C26—C31—H31C	109.5
C20—C15—C16	117.1 (2)	H31A—C31—H31C	109.5
C20—C15—C14	120.6 (2)	H31B—C31—H31C	109.5
C16—C15—C14	122.1 (2)	C29—C32—H32A	109.5
C18—C19—C20	118.7 (3)	C29—C32—H32B	109.5
C18—C19—H19	120.6	H32A—C32—H32B	109.5
C20—C19—H19	120.6	C29—C32—H32C	109.5
C18—C17—C16	119.1 (3)	H32A—C32—H32C	109.5
C18—C17—H17	120.4	H32B—C32—H32C	109.5
C16—C17—H17	120.4	C29—C33—H33A	109.5
C17—C18—C19	121.4 (3)	C29—C33—H33B	109.5
C17—C18—Cl4	120.1 (2)	H33A—C33—H33B	109.5
C19—C18—Cl4	118.5 (2)	C29—C33—H33C	109.5
N4—C1—N6	110.7 (2)	H33A—C33—H33C	109.5
N4—C1—H1	124.6	H33B—C33—H33C	109.5
N6—C1—H1	124.6	C29—C34—H34A	109.5
C4—C27—N6	118.0 (2)	C29—C34—H34B	109.5
C4—C27—C28	125.5 (2)	H34A—C34—H34B	109.5
N6—C27—C28	116.37 (19)	C29—C34—H34C	109.5
C9—C8—C7	121.1 (3)	H34A—C34—H34C	109.5
C9—C8—Cl3	119.7 (2)	H34B—C34—H34C	109.5
C7—C8—Cl3	119.2 (2)	C26—C35—H35A	109.5
C8—C9—C10	118.9 (3)	C26—C35—H35B	109.5
C8—C9—H9	120.6	H35A—C35—H35B	109.5
C10—C9—H9	120.6	C26—C35—H35C	109.5
C6—C5—C10	116.8 (2)	H35A—C35—H35C	109.5
C6—C5—C4	121.2 (2)	H35B—C35—H35C	109.5
C10—C5—C4	122.0 (2)		
			
N1^i^—Ni1—N4—C1	112.5 (2)	C20—C19—C18—C17	−0.3 (5)
N1—Ni1—N4—C1	−67.5 (2)	C20—C19—C18—Cl4	179.4 (2)
N4^i^—Ni1—N4—C1	−17 (64)	C2—N4—C1—N6	−1.0 (3)
Cl1^i^—Ni1—N4—C1	−155.7 (2)	Ni1—N4—C1—N6	166.02 (16)
Cl1—Ni1—N4—C1	24.3 (2)	N5—N6—C1—N4	1.0 (3)
N1^i^—Ni1—N4—C2	−84.0 (3)	C27—N6—C1—N4	−172.0 (2)
N1—Ni1—N4—C2	96.0 (3)	C1—N6—C27—C4	143.7 (3)
N4^i^—Ni1—N4—C2	146 (65)	N5—N6—C27—C4	−28.5 (4)
Cl1^i^—Ni1—N4—C2	7.8 (2)	C1—N6—C27—C28	−39.4 (3)
Cl1—Ni1—N4—C2	−172.2 (2)	N5—N6—C27—C28	148.4 (2)
N1^i^—Ni1—N1—C11	−88 (100)	C7—C8—C9—C10	3.3 (4)
N4—Ni1—N1—C11	37.1 (2)	Cl3—C8—C9—C10	−175.63 (19)
N4^i^—Ni1—N1—C11	−142.9 (2)	C9—C8—C7—C6	−3.6 (4)
Cl1^i^—Ni1—N1—C11	127.5 (2)	Cl3—C8—C7—C6	175.4 (2)
Cl1—Ni1—N1—C11	−52.5 (2)	N6—C27—C4—C5	−176.3 (2)
N1^i^—Ni1—N1—C12	103 (100)	C28—C27—C4—C5	7.1 (4)
N4—Ni1—N1—C12	−131.9 (2)	C6—C5—C4—C27	60.4 (4)
N4^i^—Ni1—N1—C12	48.1 (2)	C10—C5—C4—C27	−120.3 (3)
Cl1^i^—Ni1—N1—C12	−41.4 (2)	C8—C7—C6—C5	−0.2 (4)
Cl1—Ni1—N1—C12	138.6 (2)	C10—C5—C6—C7	4.1 (4)
C1—N4—C2—N5	0.8 (4)	C4—C5—C6—C7	−176.6 (2)
Ni1—N4—C2—N5	−165.7 (2)	N4—C2—N5—N6	−0.2 (4)
C11—N1—C12—N2	−0.5 (3)	C1—N6—N5—C2	−0.4 (3)
Ni1—N1—C12—N2	170.7 (2)	C27—N6—N5—C2	173.1 (2)
C12—N1—C11—N3	1.3 (3)	C8—C9—C10—C5	0.8 (4)
Ni1—N1—C11—N3	−169.85 (16)	C8—C9—C10—Cl2	178.5 (2)
N2—N3—C11—N1	−1.7 (3)	C6—C5—C10—C9	−4.4 (4)
C13—N3—C11—N1	−179.9 (2)	C4—C5—C10—C9	176.3 (2)
N1—C12—N2—N3	−0.5 (3)	C6—C5—C10—Cl2	178.0 (2)
C11—N3—N2—C12	1.3 (3)	C4—C5—C10—Cl2	−1.3 (3)
C13—N3—N2—C12	179.6 (2)	C4—C27—C28—O2	−132.6 (3)
C11—N3—C13—C14	134.6 (3)	N6—C27—C28—O2	50.7 (3)
N2—N3—C13—C14	−43.4 (3)	C4—C27—C28—C29	103.0 (3)
C11—N3—C13—C25	−50.1 (3)	N6—C27—C28—C29	−73.7 (3)
N2—N3—C13—C25	131.9 (2)	C14—C13—C25—O1	−135.0 (3)
N3—C13—C14—C15	−178.7 (2)	N3—C13—C25—O1	50.2 (3)
C25—C13—C14—C15	6.6 (4)	C14—C13—C25—C26	101.7 (3)
C19—C20—C15—C16	0.2 (4)	N3—C13—C25—C26	−73.1 (3)
Cl5—C20—C15—C16	−179.4 (2)	O2—C28—C29—C34	−177.5 (2)
C19—C20—C15—C14	−175.3 (3)	C27—C28—C29—C34	−51.5 (3)
Cl5—C20—C15—C14	5.1 (4)	O2—C28—C29—C33	64.7 (3)
C17—C16—C15—C20	−0.1 (4)	C27—C28—C29—C33	−169.3 (2)
C17—C16—C15—C14	175.4 (2)	O2—C28—C29—C32	−54.8 (3)
C13—C14—C15—C20	−124.7 (3)	C27—C28—C29—C32	71.2 (3)
C13—C14—C15—C16	60.0 (4)	O1—C25—C26—C35	−177.4 (3)
C15—C20—C19—C18	−0.1 (4)	C13—C25—C26—C35	−52.2 (3)
Cl5—C20—C19—C18	179.5 (2)	O1—C25—C26—C30	−54.5 (3)
C15—C16—C17—C18	−0.3 (4)	C13—C25—C26—C30	70.7 (3)
C16—C17—C18—C19	0.4 (4)	O1—C25—C26—C31	64.9 (3)
C16—C17—C18—Cl4	−179.3 (2)	C13—C25—C26—C31	−169.9 (3)

Symmetry codes: (i) −*x*+1, −*y*, −*z*+1.

### Hydrogen-bond geometry (Å, °)

**Table:**

*D*—H···*A*	*D*—H	H···*A*	*D*···*A*	*D*—H···*A*
O1—H1A···Cl1^ii^	0.82	2.36	3.1460 (19)	160
O2—H2A···Cl1^ii^	0.82	2.32	3.123 (2)	169

Symmetry codes: (ii) −*x*+2, −*y*, −*z*+1.
